# *Lycium barbarum* Polysaccharides Protect Human Lens Epithelial Cells against Oxidative Stress–Induced Apoptosis and Senescence

**DOI:** 10.1371/journal.pone.0110275

**Published:** 2014-10-15

**Authors:** Bing Qi, Qingshan Ji, Yuechun Wen, Lian Liu, Xiaoling Guo, Guanghui Hou, Guifang Wang, Jingxiang Zhong

**Affiliations:** 1 Department of Ophthalmology, the First Affiliated Hospital of Jinan University, Guangzhou, Guangdong, China; 2 Department of Ophthalmology, Affiliated Anhui Provincial Hospital of Anhui Medical University, Hefei, Anhui, China; 3 Department of Cell Biology, the Cell Biology Institute of Jinan University, Guangzhou, Guangdong, China; 4 Department of Ophthalmology, the Third Affiliated Hospital of Jinan University, Zhuhai, Guangdong, China; University of Pecs Medical School, Hungary

## Abstract

**Objectives:**

We aimed to investigate the protective effect of *Lycium barbarum* polysaccharides (LBPs) against oxidative stress–induced apoptosis and senescence in human lens epithelial cells.

**Methods:**

To study apoptosis, SRA01/04 cells, a human lens epithelial cell lines, were exposed to 200 µM hydrogen peroxide (H_2_O_2_) for 24 h with or without pretreatment with LBPs. Cell viability was measured using a Cell Counting Kit-8 (CCK-8) assay. Cell apoptosis, intracellular reactive oxygen species (ROS), and the loss of mitochondria membrane potential (Δψm) were detected by flow cytometric analyses. Expression levels of Bcl-2 and Bax proteins were measured by western blot analysis. The levels of malondialdehyde (MDA), superoxide dismutase (SOD), and glutathione (GSH) were quantized using commercial enzymatic kits according to the manufacturer's instructions. To study senescence, SRA01/04 cells were pre-incubated with LBPs and all cells were then exposed to 100 µM H**_2_**O**_2_** for 96 h. Cellular senescence was assessed by morphologic examination and senescence-associated β-galactosidase (SA-β-gal) staining.

**Results:**

LBPs significantly reduced H**_2_**O**_2_**-induced cell apoptosis, the generation of ROS, the loss of Δψm, and the levels of MDA. LBPs also inhibited H**_2_**O**_2_**-induced downregulated Bcl-2 and upregulated Bax proteins and increased the levels of SOD and GSH enzyme activity. Moreover, LBPs significantly attenuated H**_2_**O**_2_**-induced cellular senescence.

**Conclusions:**

These findings suggested that LBPs protect human lens epithelial cells from H**_2_**O**_2_**-induced apoptosis by modulating the generation of ROS, loss of Δψm, Bcl-2 family, and antioxidant enzyme activity and attenuating cellular senescence.

## Introduction

Age-related cataracts, also known as senile cataracts, are characterized by the gradual accumulation of cloudy deposits on the ocular lens of the elderly. Although surgery has proved effective for cataracts, it is associated with high cost and inevitable risks; therefore, cataracts remain the main cause of vision loss and blindness worldwide [1], [2]. Oxidative stress caused by reactive oxygen species (ROS) has long been recognized as the major mechanism by which cells are damaged and cataracts are formed [3]–[5]. Hydrogen peroxide (H_2_O_2_) is the main intracellular ROS in the aqueous humor that can cause protein oxidation and aggregation, lipid peroxidation, and DNA damage, and can decrease antioxidant levels in the lens, eventually accelerating the damage to the lens epithelial cells, resulting in subsequent cataract development [6]–[8]. Thus, supplementation with antioxidant nutrients is one reasonable approach to prevent cataract development.

*Lycium barbarum* is a well-known traditional Chinese herbal medicine that has multiple pharmacological and biological functions, including neuroprotection [9]–[12], antioxidant properties [13]–[15], anti-aging properties [16], [17], cytoprotection [18], [19], and immuno-modulating properties [14], [20]. *L. barbarum* polysaccharides (LBPs) extracted from *L. barbarum* fruits, are believed to be the main component responsible for these biological activities [21]. Based on the antioxidant activity of LBPs, many studies have demonstrated that LBPs have a protective effect against oxidative injury in various cells and tissues. Studies have shown that LBPs significantly alleviate exhaustive exercise-induced oxidative stress in a rat's skeletal muscle [22]. Another study found that LBPs significantly inhibited oxidative stress and improved arterial compliance in rats [23]. LBPs were also demonstrated to protect H**_2_**O**_2_**-induced breaks in the DNA in mouse testicular [24], liver, and kidney tissue from the oxidative damage caused by streptozotocin-induced diabetic rats [25]; however, it was not known whether LBPs can protect lens epithelial cells from oxidative stress.

In the current study, the ability of LBPs to protect against the adverse effects of H_2_O_2_ on apoptosis, senescence, cell viability, the generation of ROS, mitochondrial membrane potential (**Δ**ψm), pro-apoptotic proteins, and the level of antioxidant enzymes in human lens epithelial cells was assessed in vitro.

## Materials and Methods

### Preparation of LBP

*Lycium barbarum* was purchased from Ning Xia Huizu Autonomous Region, People's Republic of China. Polysaccharides from Lycium barbarum was prepared by the method of Yu [26]. The polysaccharide content of the extract was measured by phenolsulfuric method [27]. Result showed that the content of the polysaccharides in the extract may reach to 95%. The extracts were freeze-dried into powder form for storage. For experimental use, the freeze-dried powder of LBP was freshly diluted with DMEM.

### Cell culture and treatment

The SV40 T-antigen-transformed human lens epithelial cell line [28], SRA01/04 was obtained from the Cancer Institute and Hospital of the Chinese Academy of Medical Sciences (Beijing, China). Cells were cultured in Dulbecco's modified Eagle's medium (DMEM; Gibco, Grand Island, NY, USA) supplemented with 10% fetal bovine serum (FBS; Hyclone, Logan, UT, USA), 100 U/mL penicillin, and 100 mg/mL streptomycin in humidified 5% CO_2_ at 37°C. When grown to 80–85% confluence, the cells were either treated with 200 µM H**_2_**O**_2_** (Sigma-Aldrich Co., LCC, St Louis, MO, USA) for 24 h or pre-incubated with different concentrations of LBPs for 24 h and then treated with 200 µM H**_2_**O**_2_**. At the indicated time points, the cells were collected for different assays.

### Cell viability assay

The cells (1×10^4^ cells/well) were seeded into 96-well plates with five replicates for each group. The next day, the cells were pretreated with different concentrations of LBPs (0, 50, 100, 200, 400, 800, 1600 mg/L, respectively) for 24 h and exposed to 200 µM H**_2_**O**_2_**. After incubating for 24 h, the cells were inoculated with 10 µL of CCK-8 solution (Dojindo, Kumamoto, Japan) and incubated again for 2 h at 37°C, after which absorbance was measured at an optical density of 450 nm.

### Cell apoptosis assay

Annexin V-FITC/propidium iodide (PI) staining (Nanjing KeyGen Biotech. Co. Ltd., Nanjing, China) was used to quantify the amount of cell apoptosis. Briefly, SRA01/04 cells were grown on a six-well plate at 2×10^5^ cells/plate and incubated with or without 400 mg/L LBPs for 24 h before the treatment with 200 µM H**_2_**O**_2_**. Thereafter, the cells were collected and stained with Annexin V-FITC/PI in binding buffer for 20 min. The stained cells were then analyzed using the BD FACSAria flow cytometry system.

### Measurement of ROS

The intracellular ROS levels were determined using the 2′7′-dichlorofluorescin diacetate (DCFH-DA) assay kit (Beyotime Institute of Biotechnology, Nantong, China). In brief, SRA01/04 cells were pretreated with or without 400 mg/L LBPs for 24 h and then subjected to 200 µM H**_2_**O**_2_**. Four hours later, the cells were harvested and incubated with DCFH-DA (10 µM final concentration) for 30 min at 37°C in the dark. After incubation, the ROS generation was determined using the BD FACSAriaflow cytometry system.

### Measurement of Mitochondria Membrane Potential (Δψm)

**Δ**ψm was evaluated using the 5,5′,6,6′-tetrachloro-1,1′,3,3′-tetraethylbenzimi-dazolylcarbocyanine iodide (JC-1) assay kit (KeyGen Biotech. Co. Ltd., Nanjing, China). The cells were incubated with 200 µM H**_2_**O**_2_** for 24 h or pretreated with 400 mg/L LBPs for 24 h before treatment with H**_2_**O**_2_**. Then, JC-1 dye (final concentration, 5 µM) was added. After incubating for 20 min at 37°C, the Δψm of cells was examined using the BD FACSAria flow cytometry system. Mitochondrial depolarization was assessed by a decrease in the intensity ratio of the red/green fluorescence.

### Measurement of MDA, SOD, and GSH

SRA01/04 cells were pretreated with LBPs or its vehicle control for 24 h and incubated with 200 µM H_2_O_2_ for 24 h. At the end of the treatment, the cells were harvested and sonicated with phosphate buffer (pH 6.8) containing 1.0 mM phenylmethylsulfonyl fluoride to obtain cell homogenates. The homogenates were centrifuged at 4,000 rpm at 4°C for 10 min. The supernatants were used for measuring cellular MDA, SOD, and GSH using the commercially available assay kits (Jiancheng Biochemical Inc., Nanjing, China). The MDA level was calculated by evaluating the thiobarbituric acid reacting substances at a wavelength of 532 nm using the Infinite M200 microplate reader (Tecan Group Ltd., Männedorf, Switzerland). SOD activity was examined using the xanthine oxidase method as previously described [29] and absorbance was determined at 450 nm. GSH levels were measured based on the Ellman [30] method. The cell homogenate was mixed with reaction buffer (pH 8.0) and 5,5'-dithiobis-(2-nitrobenzoic acid) for 5 min. Color development was measured at 412 nm. All values were normalized according to the total protein concentration of the same sample. The results for MDA, SOD, and GSH were defined as µmol/g, U/mg protein, and mg/g protein, respectively.

### Premature senescence model and SA-β-gal staining

SRA01/04 cells were grown in DMEM medium supplemented with 10% FBS in humidified 5% CO_2_ at 37°C. When grown to 70% confluence, the cells were treated with 100 µM H**_2_**O**_2_** for 96 h or pretreated with 400 mg/L LBPs for 24 h followed by the addition of 100 µM H**_2_**O**_2_**. At established time points, SRA01/04 cells were washed with PBS, fixed in 4% paraformaldehyde for 3–5 min at room temperature, and rinsed with PBS. The cells were then incubated with freshly prepared SA-β-gal stain solution (Beyotime Institute of Biotechnology, Nantong, China) overnight at 37°C. Total and blue-stained cells were counted in 10 fields at a magnification of 40 times, and the SA-β-gal-positive cells were expressed as a percentage of total cells.

### Cell cycle analysis

PI staining kit (KeyGen Biotech. Co. Ltd., Nanjing, China) and flow cytometry were used to measure cell cycle distribution. The SRA01/04 cells were cultured in six-well plates and pretreated with LBPs or its vehicle control for 24 h and then incubated with 200 µM H_2_O_2_ for 96 h. At the end of treatment, SRA01/04 cells were collected and washed with PBS, fixed in 70% ethanol at 4°C, and treated with 10 mg/mL RNase for 30 min at 37°C. Finally, cells were stained with 50 µg/mL PI and analyzed using the BD FACSAria flow cytometry system.

### Western blot analysis

After all treatments, cultured cells were washed with cold PBS and then lysed in a RIPA buffer (Beyotime Institute of Biotechnology, Nantong, China) containing a Protease Inhibitor Cocktail (Roche Applied Science, Mannheim, Germany) and centrifuged at 12000 g for 20 min at 4°C. The supernatant protein concentration was measured using a BCA assay kit (Beyotime Institute of Biotechnology, Nantong, China) according to the manufacturer's instructions, after which 50 µg protein from the cells were subjected to 8–10% SDS-PAGE and transferred into a polyvinylidene difluoride membrane. The blot was incubated with antibody against Bcl-2, Bax (1∶1000, ProteinTech Group, Chicago, IL, USA), and GADPH (1∶3000, Cell Signaling Technology, Danvers, MA, USA). The enzyme used was horseradish peroxidase–conjugated secondary antibody (Beyotime Institute of Biotechnology, Nantong, China). Signals were visualized with enhanced chemiluminescence (ECL) (Pierce Chemical Co., Rockford, IL, USA) and quantitated using Image J. The ratio of the expression of target proteins was determined after normalizing the individual GADPH levels. Each experiment was repeated three times.

### Statistical analysis

Data were analyzed using the SPSS 16.0 (SPSS Inc., Chicago, USA). Values were presented as mean ± S.D. For comparison of the different groups, analysis of variance (ANOVA) with the post-hoc least significant difference (LSD) test was used. p<0.05 was considered statistically significant.

## Results

### LBP reduced H_2_O_2_-induced cell apoptosis in SRA01/04 cells

**Figure 1:**
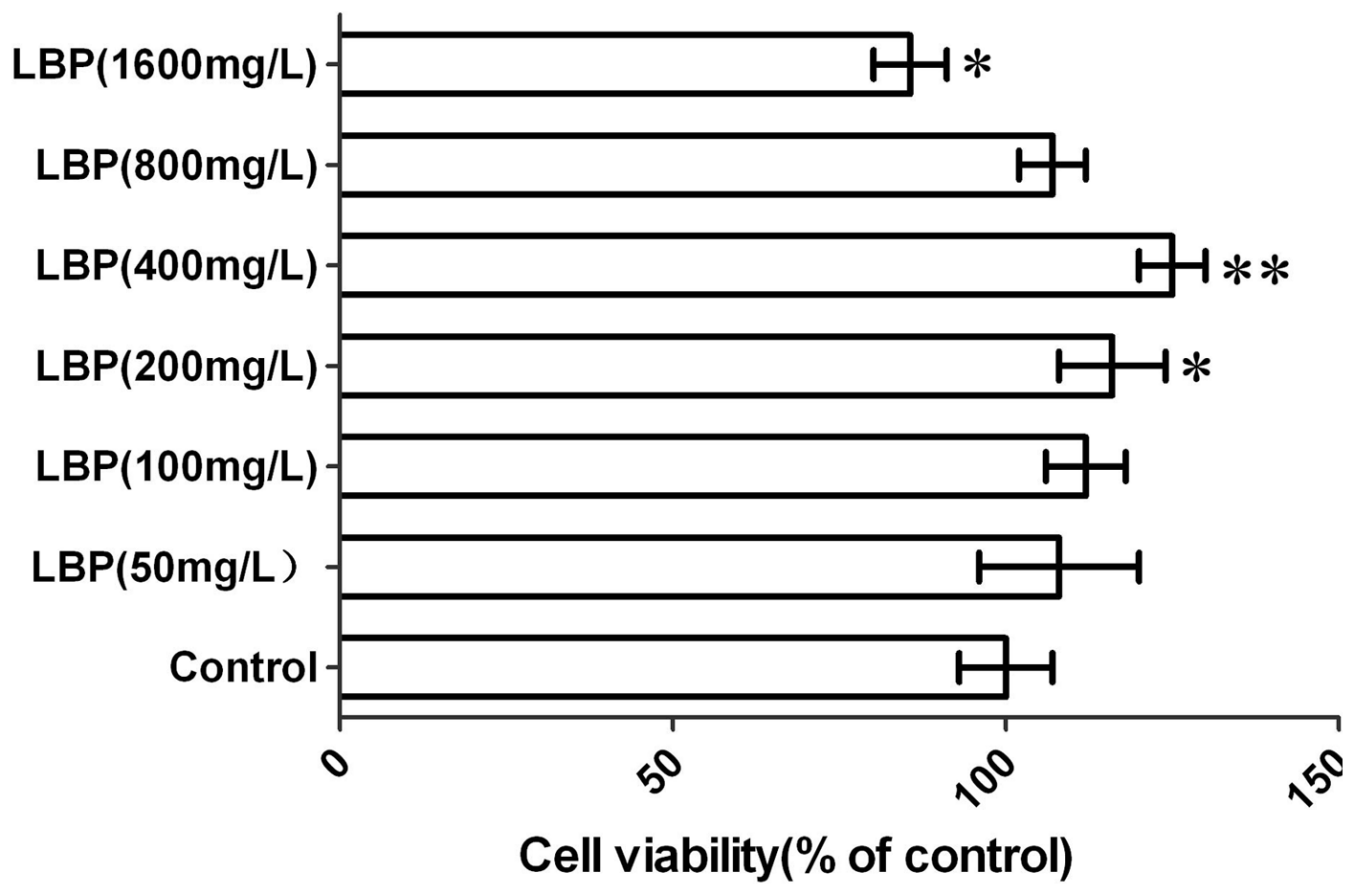
Effect of LBP on the growth of SRA01/04 cells. The SRA01/04 cells were treated with different concentration of LBPs for 24 h, and cell viability was evaluated by CCK-8 assay. Data represented the means ± SD and obtained from five independent experiments. *P<0.05, compared with control. **P<0.01, compared with control.

We selected 200 µM H**_2_**O**_2_** as the working concentration according to our preliminary experiment (data not shown). SRA01/04 cells were incubated with multiple concentrations of LBPs (0, 50, 100, 200, 400, and 800 mg/L) and showed no significant cytotoxicity; however, cell cytotoxicity was detected at a concentration of 1600 mg/L LBPs. In addition, LBPs promote SRA01/04 cell growth in a dose-dependent manner at a concentration of 200–400 mg/L (P<0.05) (Fig. 1).

**Figure 2:**
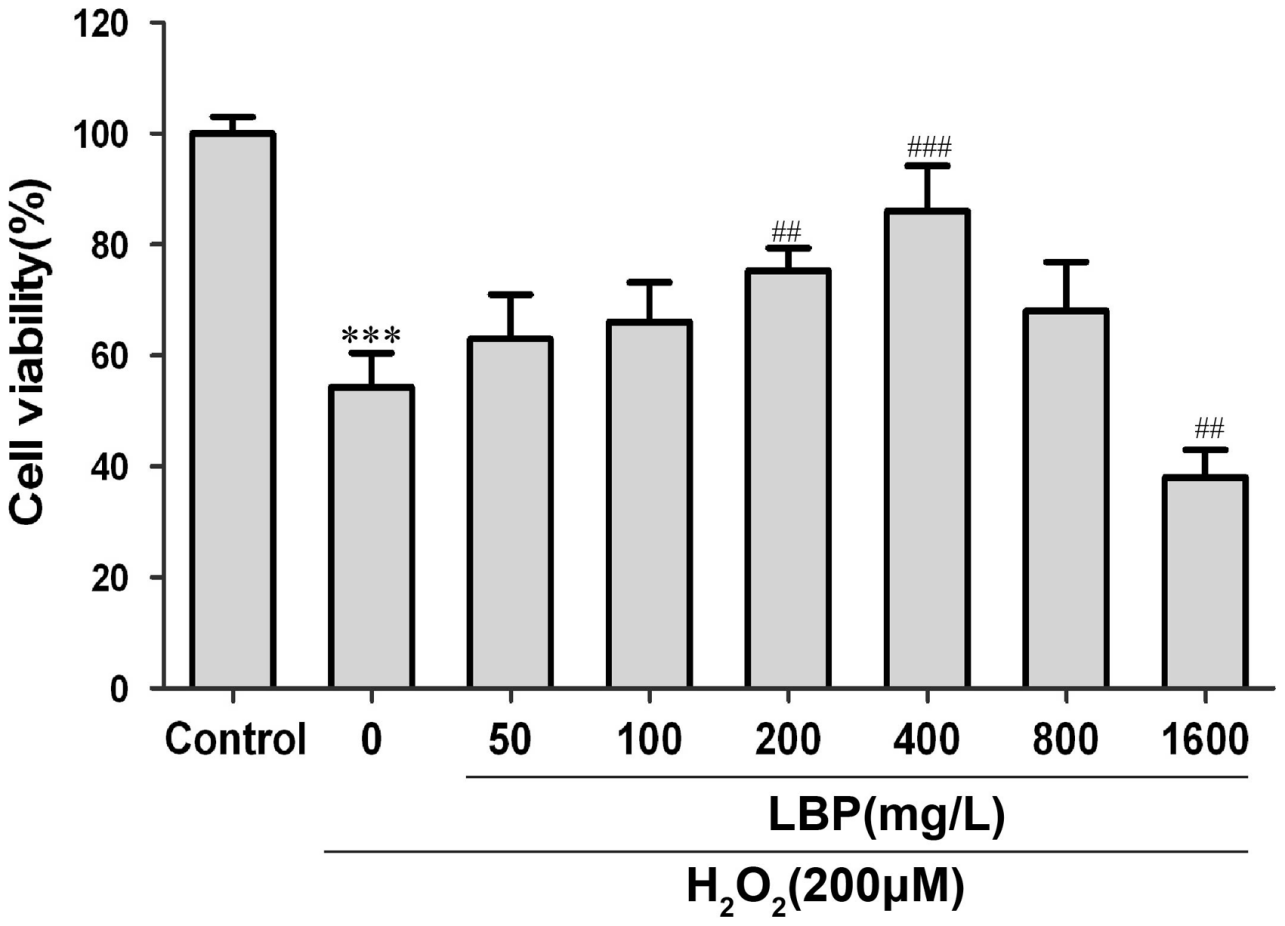
LBP increased the viability of H_2_O_2_ treated cells in an appropriate concentration. The SRA01/04 Cells were cultured with different concentration of LBPs for 24 h before incubated with 200 µM H_2_O_2_ for 24 h and cell viability was measured by CCK-8 assay. H_2_O_2_ treatment significantly decreased the viability of SRA01/04 cells, however in the presence of LBPs (concentration of 200–400 mg/L), the induction of cell death was blocked in a dose-dependent manner. Data are represented as means ± SD of five individual experiments. ***P<0.001, compared with control; ^##^P<0.01 and ^###^P<0.001, compared with H_2_O_2_ treatment alone.

To confirm LBPs protection against H**_2_**O**_2_**-induced cell damage in SRA01/04 cells, the viability of SRA01/04 cells after 200 µM H**_2_**O**_2_** incubation for 24 h, with or without LBP pretreatment at different concentrations, was detected. As shown in Fig. 2, exposure to 200 µM H**_2_**O**_2_** for 24 h reduced cell viability to 54.3±6.1%, and LBP pretreatment at a concentration of 200–400 mg/L for 24 h prevented this reduction in cell viability. LBPs exerted maximum protection at 400 mg/L.

**Figure 3:**
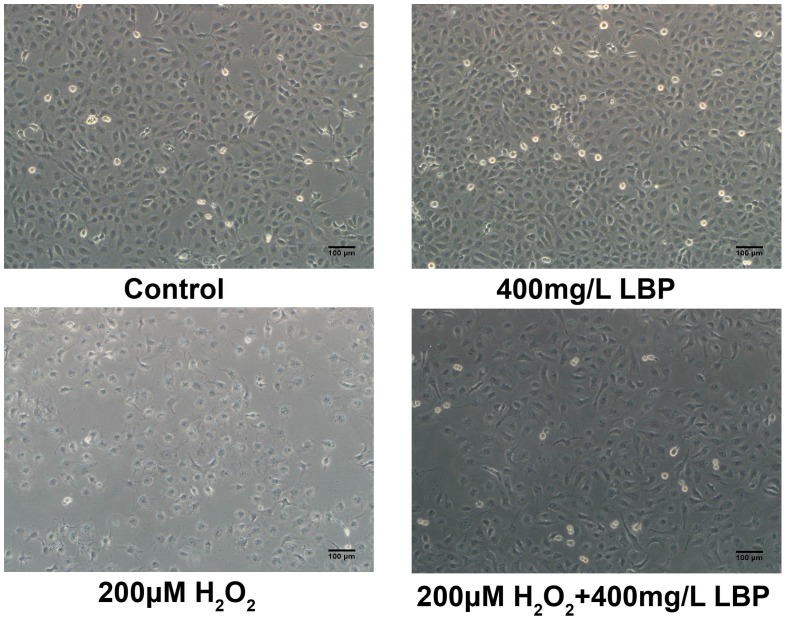
LBP inhibited morphologic changes of SRA01/04 induced by H_2_O_2_. When treated with H_2_O_2_ (200 µM) for 24 h, a large portion of cells showed signs of apoptosis such as detachment, and cytoplasmic condensation leading to rounding. However, treatment with LBPs inhibited H_2_O_2_-induced cell morphologic changes.

**Figure 4:**
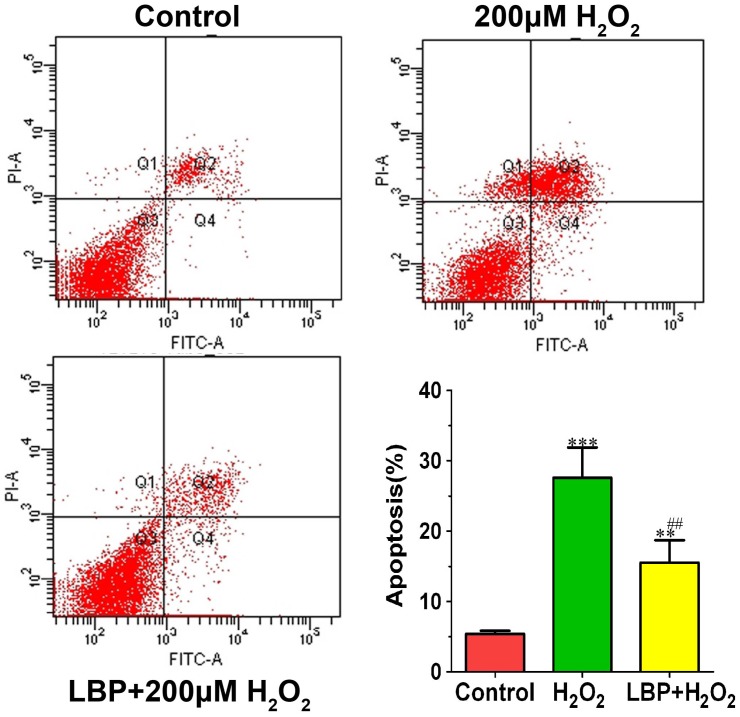
LBP reduced H_2_O_2_-induced SRA01/04 cell apoptosis. H_2_O_2_ treatment significantly increased SRA01/04 cell apoptosis. However, the proportion of apoptosis cells was significantly decreased in the LBP + H_2_O_2_ groups. Data are represented as means ± SD of three individual experiments. ^***^P<0.001, compared with control; ^##^P<0.01 compared with H_2_O_2_ treatment alone.

Next, whether LBPs would protect SRA01/04 cells against H**_2_**O**_2_**-induced cell apoptosis was examined. Based on the results from CCK-8 assay, the SRA01/04 cells were incubated with or without 400 mg/L LBPs and then subjected to 200 µM H**_2_**O**_2_** for 24 h. As observed under an inverted microscope, a high portion of cells demonstrated apoptosis-like changes include blebbing of the plasma membrane, rounding and shrinkage of cells before the plasma membrane disruption in the H**_2_**O**_2_** –alone group; however, the proportion of apoptosis-like cells decreased in the group that was pretreated with LBPs (Fig. 3). Flow cytometric analysis of the apoptotic cells also demonstrated that LBP administration (400 mg/L) led to a significant decrease in the percentage of apoptotic cells compared with that in cells treated only with H**_2_**O**_2_**. The rate of cell apoptosis was 13.8±1.25% (n = 3) in the LBP + H**_2_**O**_2_** group, much lower than that in the H**_2_**O**_2_** group (25.9±1.5%) (n = 3) (Fig. 4).

### LBP suppressed H_2_O_2_-induced the generation of ROS in SRA01/04 cells

**Figure 5:**
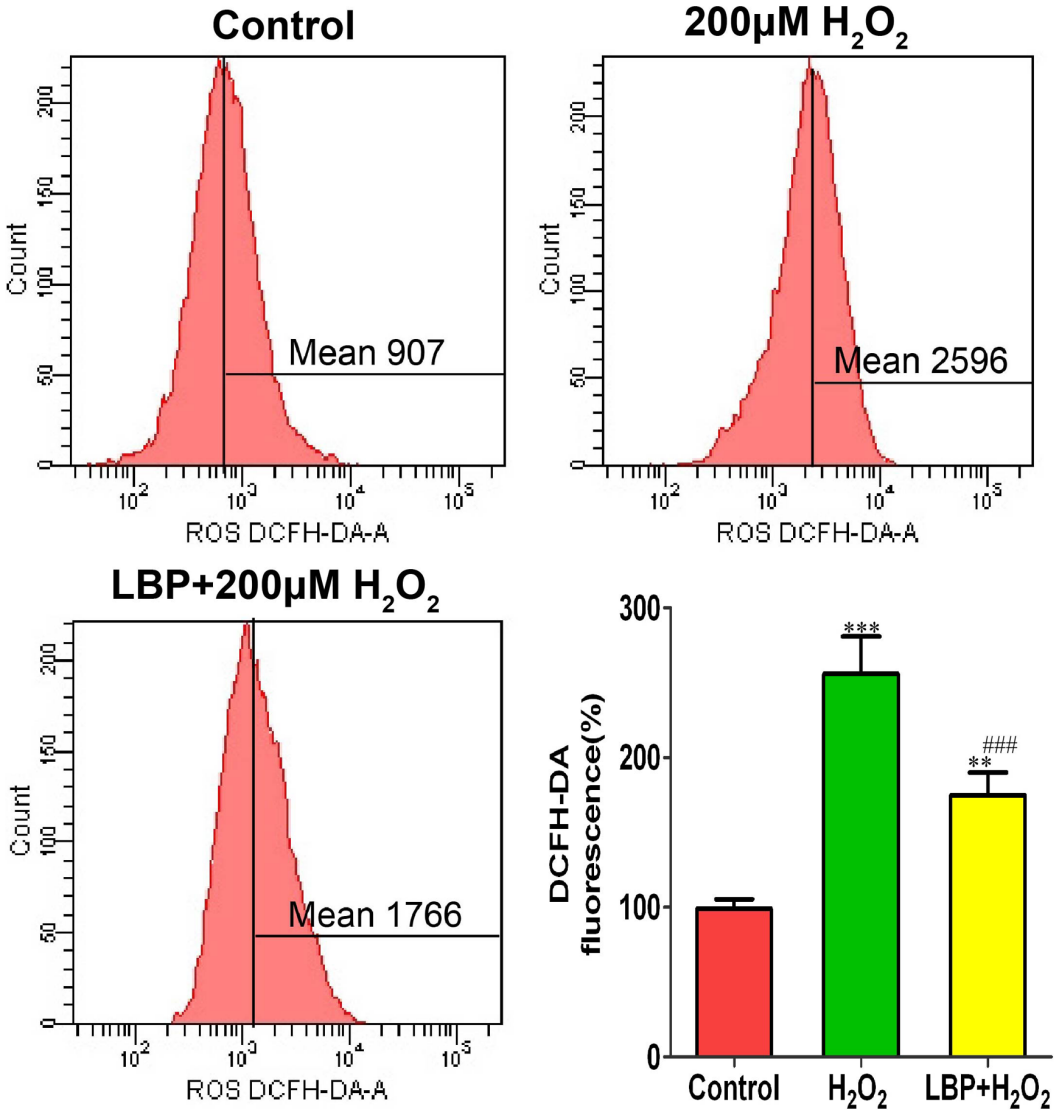
LBP suppressed H_2_O_2_-induced generation of ROS in SRA01/04 cells. The SRA01/04 cells were cultured with or without 400 mg/L LBPs for 24 h before exposed to 200 µM H_2_O_2_ for 4 h. Intracellular ROS was detected by DCFH-DA assay. Data are represented as means ± SD of three individual experiments. **P<0.01 and ***P<0.001, compared with control; ^###^P<0.001, compared with H_2_O_2_ treatment alone.

DCFH-DA was used to measure the level of ROS production. As shown in Fig. 5, treatment with 200 µM H**_2_**O**_2_** for 4 h resulted in the generation of ROS with approximately a two-fold increase compared to that in the control cells; however, pretreatment with 400 mg/L LBPs significantly decreased the levels of ROS in H**_2_**O**_2_**-exposed cells.

### LBP prevented H_2_O_2_-induced loss of Δψm in SRA01/04 cells

**Figure 6:**
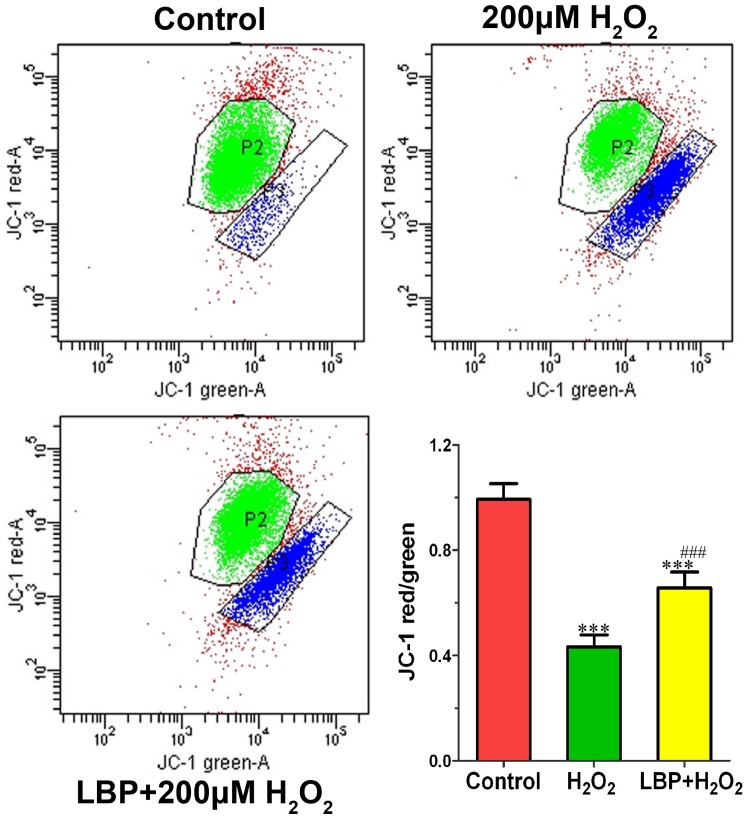
LBP stabilized H_2_O_2_-induced loss of Δψm in SRA01/04 cells. Cell were pretreated with or without 400 mg/L LBPs for 24 h, followed by exposed to 200 µM H_2_O_2_ for 24 h and stained with JC-1 dye. Data are represented as means ± SD of three individual experiments. ***P<0.001, compared with control; ^###^P<0.001, compared with H_2_O_2_ treatment alone.

Because loss of Δψm might be associated with early apoptosis, mitochondrial membrane depolarization in the H**_2_**O**_2_**-treated and LBP + H**_2_**O**_2_**–treated cells (200 µM H**_2_**O**_2_**, 24 h) was assessed using JC-1. Less than 4% of the control cells showed mitochondrial depolarization. After H**_2_**O**_2_** treatment, the percentage of cells that displayed mitochondrial depolarization increased in both but the levels were much lower in the LBP + H**_2_**O**_2_**–treated cells (Fig. 6), which suggested that LBP can protect **Δ**ψm of SRA01/04 exposed to H**_2_**O**_2_**.

### LBP inhibited H_2_O_2_-induced pro-apoptotic cell death signaling in SRA01/04 cells

**Figure 7:**
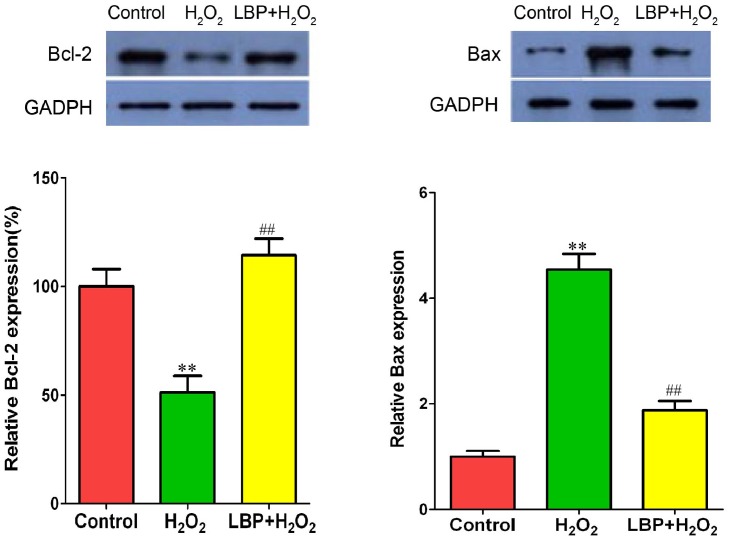
LBP inhibited H_2_O_2_-induced Bcl-2 downregulation and Bax upregulation in SRA01/04 cells. The protein expressions of Bcl-2 and Bax in H_2_O_2_ treated SRA01/04 cells were detected by Western blot analysis. LBPs significantly inhibited the H_2_O_2_-induced Bcl-2 down-regulation and Bax up-regulation in SRA01/04 cells treated with 200 µM H_2_O_2_ for 24 h. Data are represented as means ± SD of three individual experiments. **P<0.01, compared with control; ^##^P<0.001, compared with H_2_O_2_ treatment alone.

To elucidate the potential molecular mechanisms involved in the protection of LBPs following H**_2_**O**_2_** treatment, the level of apoptotic signaling molecules in the mitochondria, including Bcl-2 and Bax, was evaluated by western blot after treatment with 200 µM H**_2_**O**_2_**. Compared to the H**_2_**O**_2_**-treated cells, LBPs significantly inhibited the expression of Bax in SRA01/04 cells after treatment with H**_2_**O**_2_** for 24 h, and the expression of Bcl-2 was significantly higher in cells treated with LBPs compared to that in cells treated with H**_2_**O**_2_** alone (Fig. 7).

### LBP decreased H_2_O_2_-induced lipid peroxidation in SRA01/04 cells

**Figure 8:**
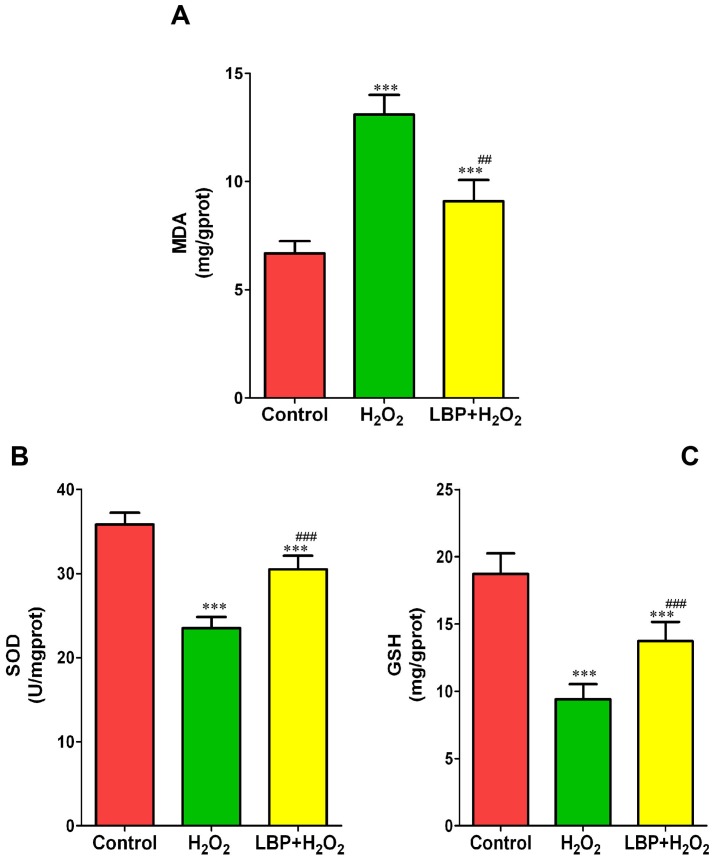
LBP alleviated H_2_O_2_-induced oxidative stress. (**A**) The MDA levels of the SRA01/04 cells exposed to 200 µM H_2_O_2_ for 24 h with or without pretreatment with LBPs. (**B**) The SOD levels of the SRA01/04 cells exposed to H_2_O_2_ (200 µM) for 24 h with or without pre-incubation with LBPs. (**C**) The GSH levels of the SRA01/04 cells exposed to H_2_O_2_ (200 µM) for 24 h with or without administration of LBPs. Data are represented as means ± SD of five individual experiments. ***P<0.001, compared with control; ^##^P<0.01 and ^###^P<0.001, compared with H_2_O_2_ treatment alone.

MDA, a lipid peroxidation product, can reflect the extent of lipid peroxidation induced by oxidative stress. As shown in Fig. 8A, compared with the control group, H**_2_**O**_2_** treatment caused a significant increase in the MDA levels; however, treatment with LBPs significantly decreased the level of MDA induced by H**_2_**O**_2_**.

### LBP alleviated H_2_O_2_-induced oxidative stress

To study the effects of LBP administration on antioxidants enzymatic in H**_2_**O**_2_**-treated cells, the activities of SOD and GSH were measured. As shown in Fig. 8B, H **_2_**O**_2_** treatment alone led to a significant decrease in SOD activity, compared with that of the control; however, pretreatment with LBPs at 400 mg/L before H**_2_**O**_2_** exposure significantly restored SOD activity in contrast to that in cells treated only with H**_2_**O**_2_**. Moreover, GSH activity presented a trend similar to that of SOD activity. GSH content in the H**_2_**O**_2_**-treated cells was also restored by LBP administration (Fig. 8C).

### LBP inhibits H_2_O_2_-induced premature senescence in SRA01/04 cells

**Figure 9:**
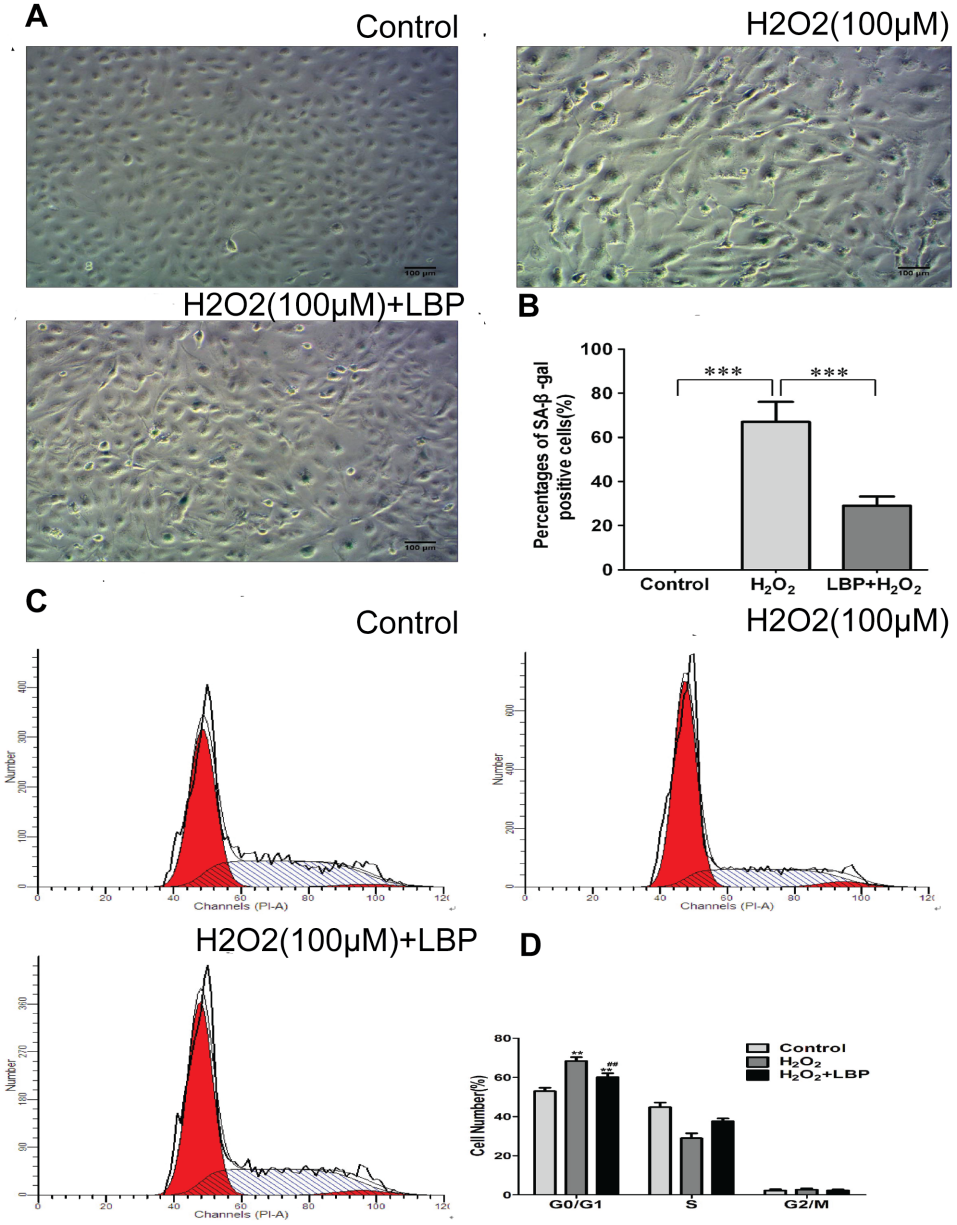
LBP alleviated H_2_O_2_-induced cell senescence in SRA01/04 cells. SRA01/04 cells were pretreated with 400 mg/L LBPs or its vehicle control for 24 h, then exposed to 100 µM H_2_O_2_ for 96 h, and subjected to SA-β-gal staining (**A**) and flow cytometric analysis (**C**). (**B**) SA-β-gal-positive cells were counted and expressed as mean ± S.D. ***P<0.001. (**D**) Cell cycle distribution is expressed as mean ± S.D. Data illustrated are representative of three independent experiments. **p<0.01, versus control; ^##^P<0.01, versus H_2_O_2_ treatment alone.

To investigate the effect of LBP on SRA01/04 cell senescence, premature senescence was induced in SRA01/04 cells by long-term treatment with low doses of H_2_O_2_. Four days after H_2_O_2_ treatment, SRA01/04 cells began to gradually exhibit a large and flattened morphology, reminiscent of cell senescence (Fig. 9A). SA-β-gal staining was then performed. As shown in Fig. 9B, H_2_O_2_ treatment resulted in a significant increase to 67±9.1% in the ratio of SA-β-gal–positive cells compared to those of the controls (P<0.001). LBPs significantly alleviated the damage caused by H_2_O_2_ and reduced the ratio of SA-β-gal–positive cells to 29±4.2% in the SRA01/04 cells.

Cellular senescence refers to a cell that no longer proliferates or is viable. It is characterized by cells exhibiting a large and flattened morphology, elevated SA-β-gal activity, and cell cycle arrest [31]–[33]. SRA01/04 cell cycle distribution was also observed in cells treated only with H_2_O_2_ or pretreated with LBP. Flow cytometric analysis revealed that the cell cycle of H_2_O_2_-treated SRA01/04 cells was arrested (P<0.01) in the G0/G1 phase; however, in the LBP-treated cells, this action was significantly attenuated in the G0/G1 phase of the cell cycle (P<0.01) (Fig. 9C–D). These observations suggested that LBPs inhibited senescence in SRA01/04 cells caused by chronic oxidative stress.

## Discussion

Many studies have demonstrated that antioxidants, such as vitamins C and E, and the carotenoid xanthophylls zeaxanthin and lutein might protect against oxidative stress in lens epithelial cells [34]–[36]. Here, we systematically investigated the antioxidant activities of LBPs and evaluated their role in H_2_O_2_-induced SRA01/04 cell damage. The results showed that treatment with LBPs effectively protected SRA01/04 cells, reduced the generation of ROS and loss of Δψm, modulated the expression of Bcl-2 and Bax proteins, and increased antioxidant enzyme activity after H_2_O_2_-induced oxidative stress.

Oxidative stress–induced apoptosis in lens epithelial cells plays an important role in cataract formation, and its prevention is of therapeutic interest. LBPs, isolated from the aqueous extracts of *L. barbarum*, have a complicated role in the life and death of cells. Ho et al. [37] investigated whether LBPs could protect neurons against homocysteine (Hcy) excitotoxicity. The results showed that LBPs significantly reduced neuronal cell death and apoptosis induced by Hcy in rat primary culture cortical neurons as detected by lactate dehydrogenase assay and caspase-3-like activity assay.

In another study [38] the protective effects of LBPs against neuronal cell death were detected in retinal ischemia/reperfusion (I/R) injuries. Pretreatment with LBPs significantly attenuated neuronal cell apoptosis in the ganglion cell layer and the inner nuclear layer of the I/R retina that was induced by surgical occlusion of the internal carotid artery. Furthermore, LBPs obviously increased the survival rate and promoted the growth of a mixed culture of rat retinal ganglion cells [39]. In the present study, we also demonstrated that LBPs significantly increase the survival of human lens epithelial cells under acute (200 µM H_2_O_2_) oxidative stress conditions. Under the inverted microscope, SRA01/04 cells exposed to H_2_O_2_ exhibited apoptotic-like signs; however, in the presence of LBPs, the proportions of apoptotic cells were significantly decreased. Moreover, flow cytometry analysis showed that LBPs markedly reduced apoptosis in H_2_O_2_-treated cells. These indicated that LBPs have a protective effect on cells by inhibiting H_2_O_2_-induced cell apoptosis.

Apoptosis is a physiological process of cell death that plays a key role in a variety of biologic systems, which has been recognized as providing an important molecular basis for cataracts [40]. The mechanisms for apoptosis involve direct damage to the mitochondria by ROS or indirect mitochondrial depolarization by pro-apoptotic Bcl-2 family proteins. In this study, H_2_O_2_-treated cells showed an increase in intracellular ROS and a loss of Δψm and unregulated the expression of Bax and downregulated the expression of Bcl-2; however, the generation of ROS was inhibited by pretreatment with LBPs. We determined that LBPs protect SRA01/04 cells against H_2_O_2_-induced apoptosis by stabilizing Δψm and increasing the expression of Bcl-2, and increasing the ratio of Bcl-2 to Bax in lens epithelial cells exposed to H_2_O_2_.

Oxidative stress results from an imbalance between ROS production and elimination. Under normal conditions, the lens contains high levels of reduced GSH [41]; abundant antioxidant enzymes [42], such as SOD, catalase (CAT), and glutathione peroxidase (GPx); and the chaperone-like functions of the crystallin proteins [43], which are likely to play an important role in protecting the lens against oxidative stress; however, when the lens is exposed to H_2_O_2_, these natural antioxidant defense systems are destroyed, and enough ROS might be generated to exceed their self-scavenging ability, resulting in oxidative stress and leading to protein modification and degradation, DNA and mitochondrial damage, and eventual cell death [44]. It was also found that the levels of GSH were significantly lower in the lens epithelial cells of patients with pseudoexfoliation (PEX) syndrome than in that of non-PEX patients [45]. LBP is well known as a powerful antioxidant and anti-aging Chinese traditional medicine. It has been proved that LBPs have a scavenging effect on hydroxyl radicals, superoxide anion, and 2,2-diphenyl-1-picrylhydroxyl free radical [46]. Yang et al. [47] investigated the antioxidant effects of LBPs on intestinal ischemia–reperfusion injury. The results suggested that LBPs treatment significantly inhibited the generation of ROS; reduced the level of MDA; and increased SOD, CAT, and GPx enzyme activities. Furthermore, studies also showed that LBPs could improve the antioxidant capacity in aged mice, increasing SOD, CAT, GSH-Px enzyme activities and reducing lipid peroxidation [48]. The protective effects of LBPs against ocular diseases have also been recently demonstrated. Pretreatment with LBPs for 1 week effectively protected the retina against neuronal apoptosis and glial cell activation through the activation of the Nrf2/HO-1 antioxidant pathway in acute ocular hypertension-induced retinal ischemia [49]. In our work, we showed that H_2_O_2_ significantly accelerated lipid peroxidation and decreased the levels of SOD and GSH enzymes; however, treatment with LBPs significantly reversed elevated MDA levels and enhanced SOD and GSH activity. The data suggested that treatment with LBPs significantly inhibited H_2_O_2_-induced lipid peroxidation, which might be related to the increased activity of antioxidant enzymes.

It has been recognized that accumulation of oxidative stress will contribute to the aging processes [50], [51]. Hence, antioxidants play an important role in slowing down biological aging. The anti-aging property of LBPs has been investigated using different models. For example, LBPs have been shown to recover anti-decrepit effects in heart and brain tissues in mice by increasing SOD activity [52]. Another aging study model investigated the life span of *Drosophila* [53] and found, surprisingly, that LBPs can significantly prolong its life span. Because oxidative stress-induced aging is one of the important risk factors for age-related cataracts, we next assessed whether LBPs play a role in the senescence of lens epithelial cells when subjected to chronic oxidative stress. We found that low doses of H_2_O_2_ treatment caused a morphological change in the cells, positive SA-β-gal staining, and G0/G1 phase cell cycle arrest, which suggest that a low dose of H_2_O_2_ treatment could induce cell senescence in SRA01/04 cells; however, LBP pretreatment dramatically reduced cellular senescence markers under chronic oxidative stress in these cells, which is consistent with a protective effect and might help explain part of the mechanism by which the life span of the cells is extended.

In conclusion, our results demonstrated that LBPs have the potential to protect human lens epithelial cells from oxidative-stress damage by modulating the generation of ROS, loss of Δψm, the Bcl-2 family, and antioxidant enzyme activity and by attenuating cell senescence; therefore, LBPs are a potent antioxidant and anti-aging agent that could be exploited as a potential implication in the prevention of cataractogeneis.
